# Mediterranean California’s water use future under multiple scenarios of developed and agricultural land use change

**DOI:** 10.1371/journal.pone.0187181

**Published:** 2017-10-31

**Authors:** Tamara S. Wilson, Benjamin M. Sleeter, D. Richard Cameron

**Affiliations:** 1 U.S. Geological Survey, Western Geographic Science Center, Menlo Park, California, United States of America; 2 U.S. Geological Survey, Western Geographic Science Center, Tacoma, Washington, United States of America; 3 The Nature Conservancy, San Francisco, California, United States of America; College of Agricultural Sciences, UNITED STATES

## Abstract

With growing demand and highly variable inter-annual water supplies, California’s water use future is fraught with uncertainty. Climate change projections, anticipated population growth, and continued agricultural intensification, will likely stress existing water supplies in coming decades. Using a state-and-transition simulation modeling approach, we examine a broad suite of spatially explicit future land use scenarios and their associated county-level water use demand out to 2062. We examined a range of potential water demand futures sampled from a 20-year record of historical (1992–2012) data to develop a suite of potential future land change scenarios, including low/high change scenarios for urbanization and agriculture as well as “lowest of the low” and “highest of the high” anthropogenic use. Future water demand decreased 8.3 billion cubic meters (Bm^3^) in the lowest of the low scenario and decreased 0.8 Bm^3^ in the low agriculture scenario. The greatest increased water demand was projected for the highest of the high land use scenario (+9.4 Bm^3^), high agricultural expansion (+4.6 Bm^3^), and high urbanization (+2.1 Bm^3^) scenarios. Overall, these scenarios show agricultural land use decisions will likely drive future demand more than increasing municipal and industrial uses, yet improved efficiencies across all sectors could lead to potential water use savings. Results provide water managers with information on diverging land use and water use futures, based on historical, observed land change trends and water use histories.

## Introduction

California has one of the most highly engineered and complex water supply and delivery systems in the world. This system must currently support a population of nearly 38 million people and one of the most productive agricultural regions found anywhere on the globe. Most surface water supplies come in the form of winter precipitation in the mountainous north and east, which is captured and redistributed to the west, central, and southern parts of the state. Recent trends show declines in winter precipitation falling as snow and reduced snowpack [1–3], decreased snow water equivalents [4], earlier spring snowmelt [5–7], and widespread changes in surface hydrology [8]. Recent declines in snowpack and shifts in snowmelt runoff have been attributed to increasing temperatures [3]. Given projected climate change, California’s water future is uncertain. Ensemble analysis of general circulation models shows a continued warming trend in coming decades while the precipitation story is less clear [9]. Some research indicates a high probability of longer, more severe droughts this century [10], may challenge water supply reliability.

The recent drought, which began back in 2012, was the most severe recorded in both the historical and the paleo-climate record [11], although century scale droughts of lower severity have been documented in the last 1200 years [12]. Most urban residents were modestly impacted by the drought, due to the 25 percent reduction in municipal water use mandated by the state in April, 2015 [13]. Despite continued drought conditions, municipal restrictions were lifted in May 2016, leaving individual water districts with the task of maintaining sustainable water use levels, depending on their unique vulnerability. By July 2016 the entire state was still gripped by varying degrees of drought severity [14] and by October 2016, officials were reporting water conservation rates had dropped nearly 10% over the prior year. With rainfall and snowpack totals in excess of 150% above average this winter (2016/2017) and reservoirs in the northern part of the state now at or near capacity, water conservation will likely continue to decline. Yet, despite heavy rains, 23% of California is still classified in some state of drought (i.e. 23.5% “abnormally dry,” 8.2% “moderate drought,” and 1% “severe drought”) as of April 14, 2017 in an area home to an estimated 10,293,138 people [14].

Municipal water use is relatively small compared to agricultural consumption, estimated at roughly 80 percent of total water use statewide and predominantly dependent on irrigation. This leaves farmers particularly vulnerable to drought in a region with a dry growing season. Satellite imagery analysis showed 626,000 more acres fallow in 2015 than in 2011 [15] resulting in $2.7 billion in lost agricultural revenue and 21,000 lost jobs [16]. Droughts in California usually result in decreased surface water rights allocations, offset by dramatic increases in groundwater pumping at unsustainable rates [17–19]. Groundwater overdraft has become common in some areas of California’s Central Valley, where many low income farming communities rely solely on local wells for drinking water, which have run dry [16]. Such water stress can potentially drive regional insecurity which can lead to regional unrest according to the United States government [20]. With a population expected to reach over 52 million people by 2060 [21] (+ 38% over 2012 population [22]) and a $54 billion dollar agriculture industry which has been shifting rapidly towards higher value tree, nut, and vine crops in recent decades [23–27]–crops less resilient to prolonged dry periods—California’s water needs will likely continue to rise.

Land use scenario research has seen tremendous growth in the last decade, with global [28], continental [29,30], national [31–37], and regional [38–41] LULC projections now available. Land use projections have been used to assess potential impacts of land change on biodiversity [34,42–47], protected areas [38,48–50], ecosystem services [51,52], wildfire risk [41], watershed management [53], carbon sequestration [54,55], and water demand [56]. Future LULC projections enable researchers and managers to visualize a wide range of potential, plausible futures outcomes for mitigation and resource planning. Scenario projections represent plausible future conditions based on historical land change trends and should not be considered absolute outcomes.

We used the Land Use and Carbon Scenario Simulator (LUCAS) [39,55,56] state-and-transition simulation model (STSM) [57,58] to project future land change and water demand scenarios from 2012 to 2062 for Mediterranean California. We developed a broad suite of future land use and land cover (LULC) scenarios based on sampling from historical, empirical land change data, representing a wide range of potential LULC and water demand futures. These include scenarios of high and low agriculture, high and low development, and a business-as-usual scenario as well as combined highest of the high and lowest of the low anthropogenic land use. We relied on historical land change and water use data for our “baseline” (1992–2012) period. For each scenario we modeled spatially explicit (1 km^2^) changes in annual LULC as well as land-use related water demand across 40 Monte Carlo simulations. This research builds on previous work which modeled future land use related water demand under a business-as-usual (BAU) scenario [56]. Efforts to quantify California’s potential future water demand are extremely important given increasing population pressures, shifts towards higher value, more intensive agriculture (i.e. perennial cropland), and the often overlooked ecosystem services provided by open space and rangelands [51,59]. Results also provide water managers and policy makers with information on diverging land use and water use futures, helping better inform land and resource management decisions.

## Materials and methods

The LUCAS model was used to model future land use scenarios and associated land-use related water demand. The LUCAS model is a form of state-and-transition-simulation model (STSM) capable of performing stochastic, Markov chain based simulations. The LUCAS model is of great utility to the land use modeling community for projecting future landscape conditions based on historical land change [38,39,55]. Its architecture is based on ST-Sim, a commonly used STSM platform available online [60] and proven model for projected landscape level change, testing mitigation strategies, and capturing model uncertainty with Monte Carlo simulations [58]. Our goal was to examine alternative water demand futures by the year 2062 and how various land use intensities may influence that demand.

### Scales and state variables

**Fig 1:**
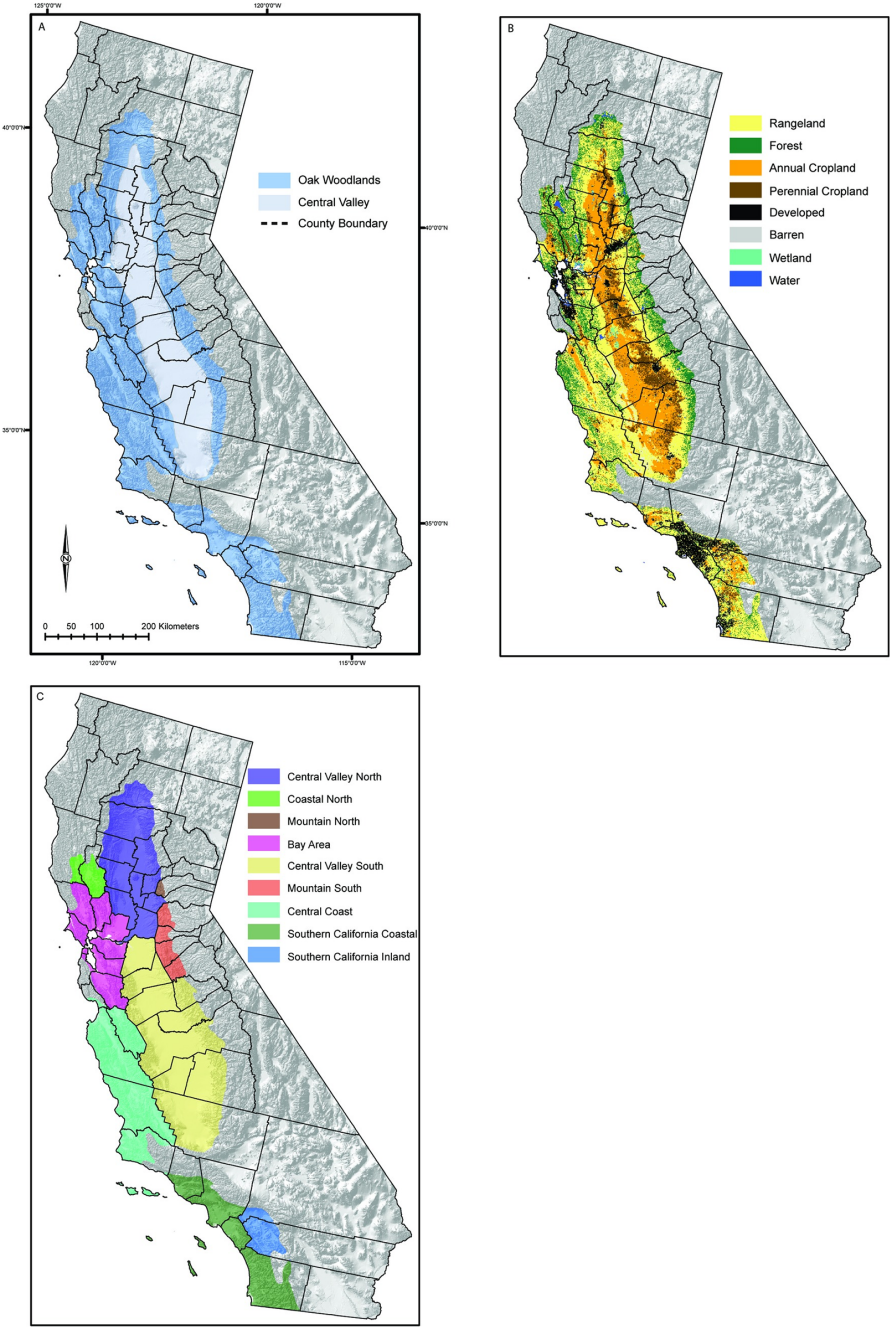
The Mediterranean California study region. (A) The Oak Woodlands and Central Valley ecological regions and 46 counties. (B) Land use and land cover (LULC) in 1992 at model onset. (C) Aggregated regions for water demand reporting. Shaded relief map from the U.S. Geological Survey’s National Atlas of the United States [64].

Our study area encompasses the Mediterranean California region of the state, divided up into two distinct, but overlapping, spatial strata. These include the California Central Valley (i.e. ‘Central Valley’) and the Central California Foothills and Coastal Mountains (i.e. ‘Oak Woodlands) ecoregions [61] and the 46 associated counties contained therein (Fig 1A). Ecoregions are commonly used in land use and land cover change research [62,63] as they represent areas with common biotic, abiotic, aquatic, and bio-geophysical attributes. Counties are more commonly used spatial units for data collection and dissemination on cropland trends, land use change, and water use.

The study area was divided into 1-km x 1-km simulation cells covering 146,410 km^2^ of land area. Simulations were run for 70 years, from 1992 initialization out to 2062. This allowed for a 20-year historical “spin-up” for model validation and a 50 year projection period. For each scenario, 40 Monte Carlo iterations were simulated.

**Fig 2:**
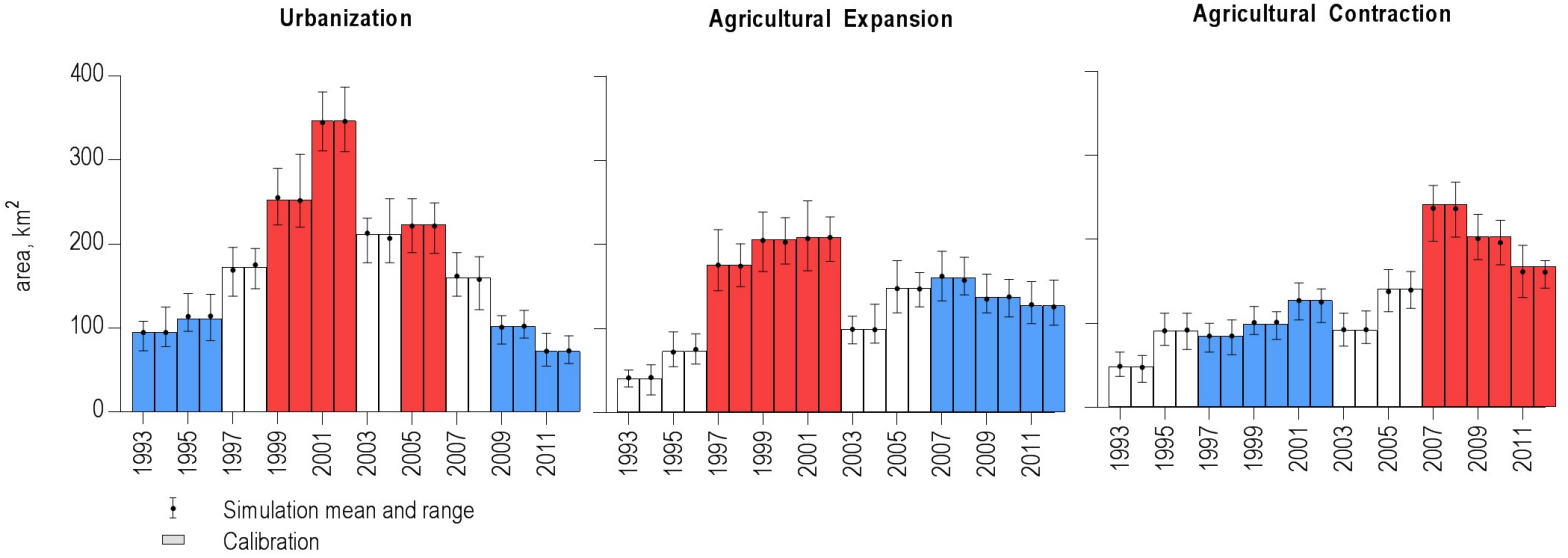
Model validation results over the historical period (1992–2012). Comparison of historical rates (1992–2012) of urbanization, agricultural expansion, and agricultural contraction (bars) in Mediterranean California derived from the Farmland Mapping and Monitoring Program data [24] with LUCAS model estimates across the 40 Monte Carlo simulations of the model (whiskers with mean, min, and max) modified from Wilson, et. al [56]. High and low rates for of each LULC transition type are indicated by red and blue bars, respectively. For agricultural expansion and contraction, high and low years are interdependent (e.g. high agriculture years defined where agriculture expansion exceeds agricultural contraction).

The LUCAS STSM tracked the following three state variables: 1.) state class type, 2) age, and 3) time-since-transition (TST). State class type is determined by each cells associated LULC type, ecoregion, and county. The landscape was classified into 8 primary LULC state classes (rangeland, forest, annual cropland, perennial cropland, developed, barren, water, wetland; Fig 1B) and 2 secondary state classes (protected rangeland and protected forest). Age and TST were tracked for the perennial agriculture state class, to model orchard/vineyard removals. Fig 1C shows aggregated areas for model output post-processing to report regional water demand change.

### Model formulation

The model was formulated to simulate changes in state variables associated with urbanization, agricultural expansion and contraction, changes within agricultural classes, and orchard removal. The ordering of transitions was randomized in each timestep and Monte Carlo iteration.

#### Land change data sources

We used historical land change data from Wilson et al. [56] which was compiled by the Farmland Mapping and Monitoring Program (FMMP) [65,66] using manual interpretation of aerial photographs bi-annually from 1992–2012 (Fig 2). The FMMP dataset includes county-based information on transitions between urban/built up lands, farmland, and grazing land (i.e. rangeland). The FMMP classes were cross-walked into our 8 primary LULC classes and form the historical baseline (1992–2012) of land change data sampled in distinct ways for each new LULC scenario. The 1992 baseline areal extent of protected rangeland and protected forest was defined by the Protected Areas Database for the United States (PAD-US) [67] and the California Protected Areas Database (CAL-PAD) [68]. Protected area establishment dates from PAD-US and CAL-PAD were used to calculate a 78 km^2^ annual rate of protection for the historical period. Agricultural statistics data were used to derive a 100 km^2^/yr-^2^ (standard deviation of 50 km^2^) transition target for the conversion of annual to perennial cropland across all scenarios. Both protection and annual to perennial cropland conversions were applied at the ecoregion level.

### Transition probabilities

The following rules were held constant across scenarios to account for orchard removal. First, all initial perennial cropland was assigned a random age, as no data exist for perennial cropland age between 1 and 45 years. The model then tracks the time since transition (TST) for each perennial cropland cell to determine orchard removal and transitions from perennial to annual cropland. On average, California orchards are removed every 25 years [69] and vineyards can remain longer. Therefore, we set the minimum orchard removal age to 20 years. For each Monte Carlo simulation and timestep, the annual transition probability for perennial cropland between 20 and 45 years of age was sampled from a cumulative transition probability of 0.95, representing age-based transition probabilities of 0.0228 and 0.0950 respectively. Perennial cropland removal is assumed to be followed by replanting of perennial cropland, therefore the state class remains unchanged and the age is reset to 0. We set the probability of 5% for perennial to annual cropland conversion within 1 year of orchard removal. In addition, perennial cropland must reach a 20 year maturity before qualifying for conversion to rangeland or annual cropland.

#### Spatial multipliers

We utilized LULC transition-specific spatial multipliers to guide the placement of projected future land change. Spatial multipliers use a probabilistic raster surface to either allow or prevent the occurrence of specified LULC conversions. Transitions into development were set to 0 for lands identified by the U.S. Geological Survey’s Protected Areas Database [67] classified as GAP Status 1, 2, and 3 and all agricultural conservation lands [70,71]. Land conversion into agricultural was set to zero probability on GAP Status 1 and 2. California Rangeland Conservation critical and priority conservation areas [72] were used to guide placement of future protected lands at the continued historical rate of 78 km^2^/yr.

Spatial adjacency rules were also defined for transitions into urbanization as well as agricultural change, expansion, and contraction. For each transition type, the probability of a cell changing into a different state class was a calculated as a function of the proportion of neighboring cells also classified as the “transitioning to” state class. For example, if none of the neighboring cells are developed the probability of a cell experiencing urbanization is zero, whereas the probability of urbanization increases with the higher the amount of neighboring developed cells.

### State attributes

Water use values were calculated for each county following Wilson et al. [56] and are summarized below. For the developed class, average water use was calculated from the U.S. Geological Surveys Water Use data for the year 2010 [73]. County-level values for ‘public supply-freshwater’ and ‘industrial self-supplied’ were summed and divided by total developed land area, calculated using the aggregated developed land use classes in the 2011 NLCD [74]. This resulted in a developed water use value in m^3^/km^2^. Applied water use for the annual and perennial cropland classes were derived from the California Department of Water Resources (CDWR) [56]. The U.S. Department of Agriculture’s 2010 Cropland Data Layer [75] was used and logically reclassified into CDWR cropland classes, which were then collapsed into annual and perennial cropland types to get an area-weighted average applied water use for each county and cropland type. Resulting water use values for each land use class were held constant and do not assume future conservation, improved efficiencies, or technological advancements. All future water demand projections were driven by varying scenarios of land use change, as our approach ties water demand to unit area.

### Model initialization

The LUCAS model runs begin with an initial state class map (i.e. LULC) and spatial strata maps (i.e. ecoregions and counties), which allows for reporting across LULC type and spatial scale. For each LULC type, transition pathways are defined which control how, where, and when (e.g. age) a LULC state class can experience an allowable transition. For example, in our model, areas designated as developed, cannot transition out of developed. Losses of developed land have not been documented in late 21^st^ century land change analyses from the region [76,77].

Our model initial conditions were based on a modified 1992 National Land Cover Dataset (NLCD) 30-meter product [78], resampled to 1 kilometer. The original 20 LULC classes were aggregated and re-classified into the following 10 LULC classes: 1) water, 2) developed, 3) barren, 4) rangeland, 5) protected rangeland, 6) forest, 7) protected forest, 8) annual cropland, 9) perennial cropland, 10) wetland (Fig 1). The protected forest and grassland classes from the 1992 NLCD were identified using the Protected Areas Database of the U.S [67] and the California Protected Areas Database [68].

### Scenario simulations

We developed a broad range of potential LULC scenarios using the baseline FMMP land change information, building on previous work which modeled a business-as-usual (BAU) scenario [56] across 40 Monte Carlo simulations (Fig 2). Scenarios include a newly run BAU scenario, high agriculture (HA), low agriculture (LA), high urban (HU) and low urban (LU), highest of the high (HH), and a lowest of the low (LL) scenarios. All scenarios model historical land change using direct transition target data for the years 1992–2012. For the projected years (2013–2062), the historical dataset was sampled from in variety of ways to generate 7 LULC scenarios described in Table 1. For the BAU scenario, in each timestep and Monte Carlo a single historical year was sampled including all transition across every county, preserving the spatial and temporal change drivers. Sampling for the HA and LA scenarios used the historically low and high years (Fig 2; Table 1) for agricultural expansion and agricultural contraction, while sampling randomly for urbanization transitions across the full historical record. This approach preserves covariance between both agricultural transition groups across space and time for a given sample year. Conversely, the HU and LU scenarios also preserve temporal and spatial variability in the historical sample year selected for urbanization rates only, sampling independently for agricultural change (i.e. 1 sample year selected and all agricultural transitions utilized) for each projected year and Monte Carlo. The LL and HH scenarios sampled independently between transition type, year, and county. For each county, transition rates for each transition type were ranked from highest to lowest and sampled accordingly (Table 1). For example in the HH scenario, the highest 20% of values for urbanization and agricultural contraction were used along with the lowest 20% of values for agricultural contraction.

**Table 1:** Land use and land cover scenario groups and associated sampling options for each transition type from the historical (1992–2012) record for Mediterranean California.

Scenario	Description	Urbanization	Agricultural Expansion	Agricultural Contraction	Covariance
**Business-As-Usual(BAU)**	Random sampling from historical data. One historical year is selected and all transitions, for each county, are selected for that year.	**1992–2012**			Preserved across county and transition.
**Low Agriculture(LA)**	All agriculture transition rates randomly sampled from historical years where low agricultural contraction was greater than agricultural expansion. Urbanization rates randomly sampled from full historical record.	1992–2012	Random sampling from 2007–2012		Preserved across county and transition.
**High Agriculture(HA)**	All transition rates randomly sampled from historical years where agricultural expansion was greater than agricultural contraction.	1992–2012	Random sampling from 1997–2002		Preserved across county and transition.
**Low Urbanization(LU)**	All transition rates randomly sampled from historical years with low urbanization.	Random sampling from 1993–1996 and 2009–2012	1992–2012		Preserved across county and transition.
**High Urbanization(HU)**	All transition rates randomly sampled from historical years with high urbanization rates.	Random sampling from the years 1999–2002 and 2005–2006	1992–2012		Preserved across county and transition.
**Lowest of the Low (LL)**	Historical transition data values ranked by county and sampled from the lowest 20% of values for urbanization and agricultural expansion and highest 20% of values for agricultural contraction for each projected time step and iteration.	Random sampling from lowest 20% of values for each county	Random sampling from lowest 20% of values for each county	Random sampling from highest 20% of values for each county	Only for each transition type at a given iteration and time step. No spatial or temporal covariance.
**Highest of the High(HH)**	Historical transition data ranked by county and sampled from the highest 20% of values for urbanization and agricultural expansion and lowest 20% of values for agricultural contraction for each projected time step and iteration.	Random sampling from highest 20% of values for each county	Random sampling from highest 20% of values for each county	Random sampling from lowest 20% of values for each county	Only for each transition type at a given iteration and time step. No spatial or temporal covariance.

**Fig 3:**
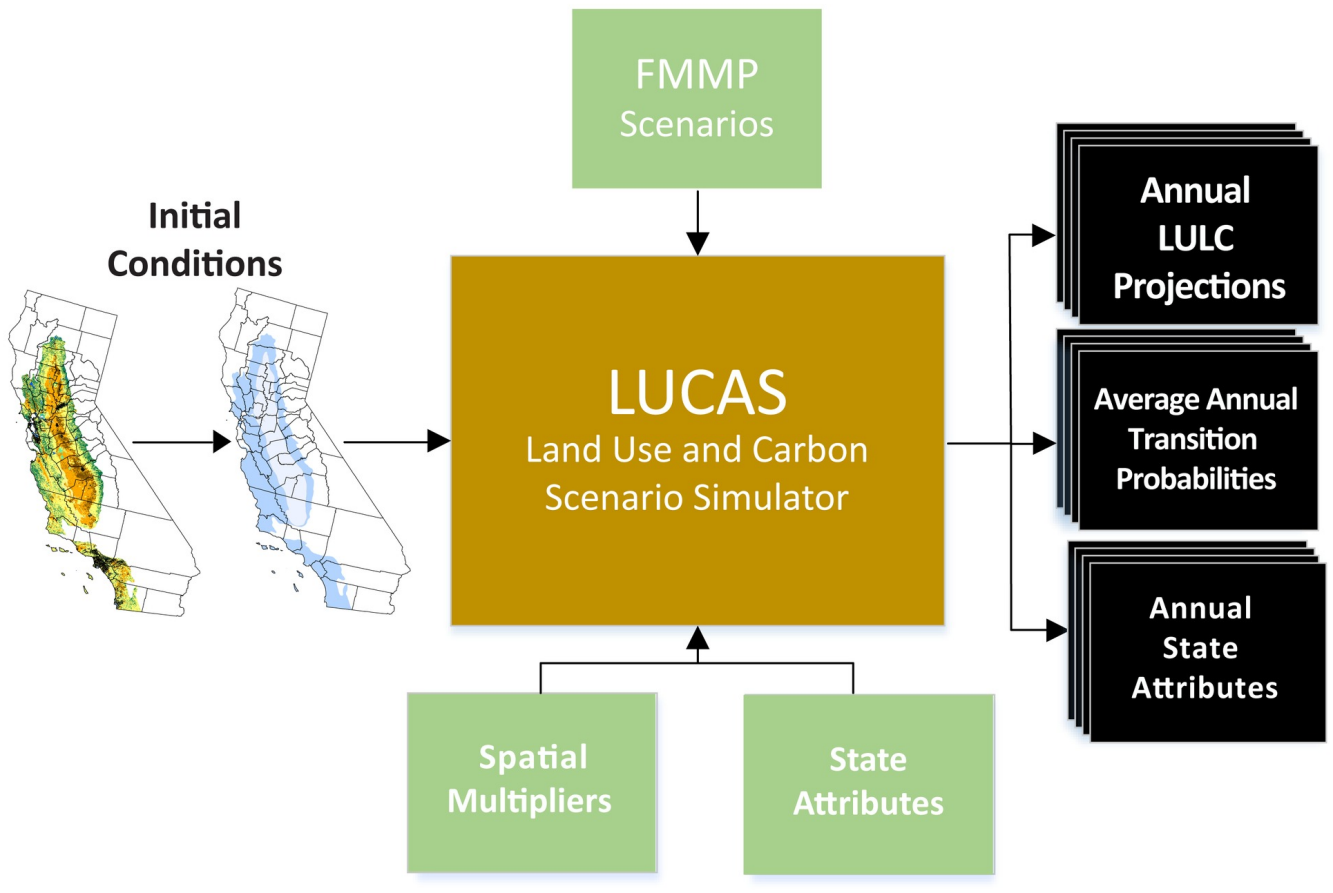
Schematic diagram of the Land Use and Carbon Scenario Simulator (LUCAS) model. Diagram shows model inputs and outputs, including the spatial initial conditions, the county and ecoregion spatial strata, Farmland Mapping and Monitoring Program (FMMP) derived scenarios spatial multipliers, state attributes (i.e. water), and model outputs.

This suite of scenarios was developed to best capture divergent potential LULC futures while representing a broad range of water use outcomes. LULC change variability among counties is maintained across both space and time in all but the HH and LL scenarios, where high and low values were ranked for each county, to reveal outcomes disaggregated in space and time. Historical annual land protection rates (78 km^2^/yr) were applied in each scenario projection as well as preservation of existing conservation farmland [71]. The specifics of each scenario are outlined in Table 1. Full model components and processes are outlined below and shown conceptually in the schematic diagram in Fig 3.

### Model validation

Fig 2 shows model validation results for each state class transition group with LUCAS model mean closely tracking the empirical transition data with model estimates ranging slightly higher or lower the observed data across the 40 Monte Carlo simulations. This validates that the LUCAS model was structurally capable of reproducing the historical transition area for each transition group. As in previous work, a pixel by pixel validation of the model was not possible due to the lack of spatially explicit reference condition data. Our 1992 NLCD initial conditions cannot be compared to later NLCD versions given mapping and classification differences. Trends in agricultural change compare favorably with estimates from the National Agricultural Statistical Service data for the 1992–2009 period [23–27]. Given collapsing of cropland types into the annual and perennial classes, a true cropland validation is not plausible, however overall modeled trends were consistent with statistical estimates. For additional model validation results, see Wilson et al. [56].

## Results

### Land-use change

**Fig 4:**
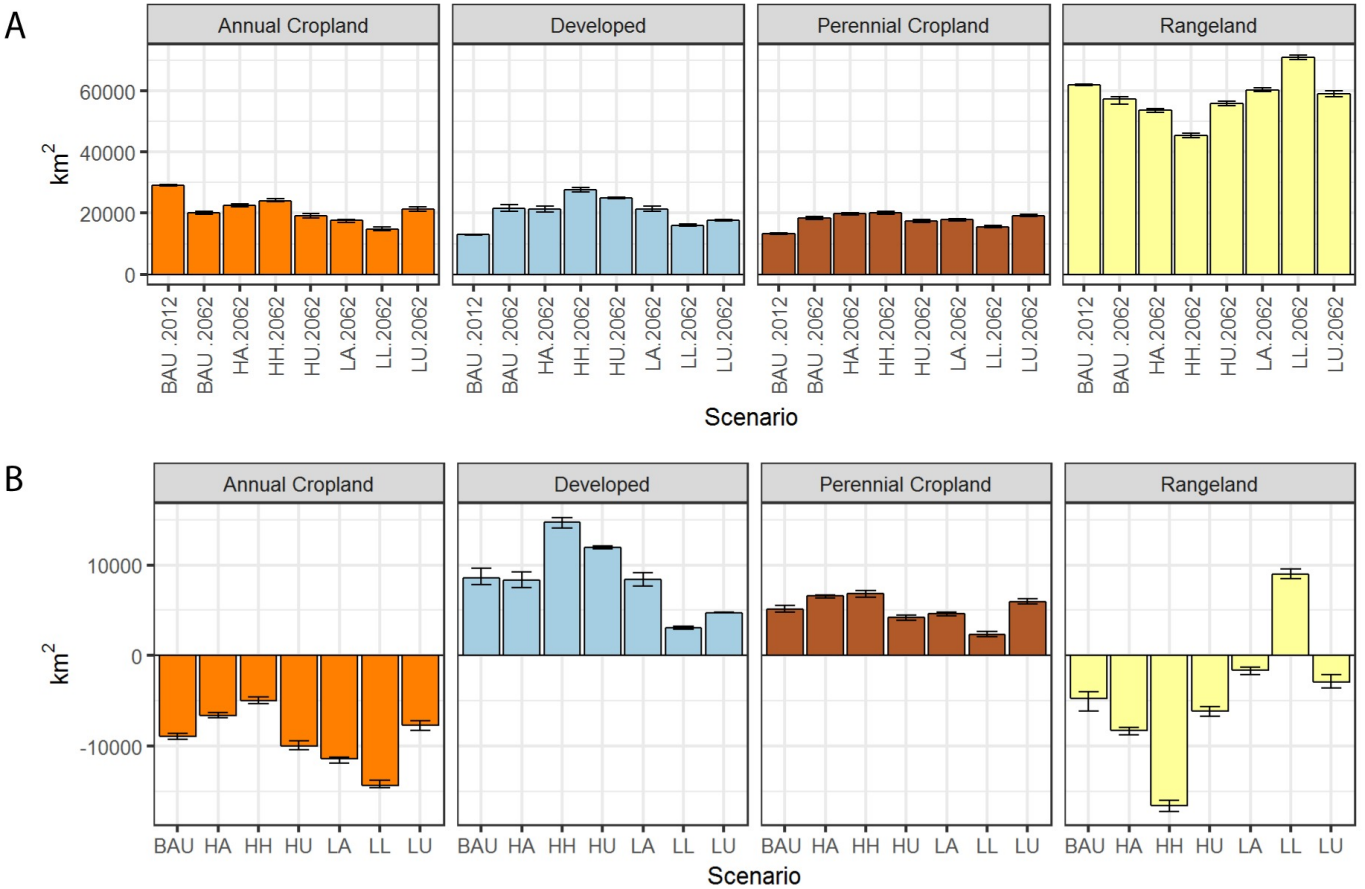
Projected land use and land cover (LULC) in Mediterranean California by 2062. (A) Change in LULC from 2012 under business-as-usual (BAU) and in 2062 for the BAU, high agriculture (HA), highest of the high (HH), high urban (HU) low agriculture (LA), lowest of the low (LL), and low urban (LU) scenarios. Bars represent the mean LULC and maximum and minimum values across 40 Monte Carlo simulations. (B) Net change in land use and land cover (LULC) from 2012–2062 with the mean (bar) and maximum and minimum values across 40 Monte Carlo simulations.

This broad suite of scenarios for future urbanization, agricultural expansion, and agricultural contraction captured a wide range of potential futures as well as the inherent uncertainty in scenario projections. For each of the 7 scenarios, annual cropland declined from 2012 to 2062, while perennial cropland and developed land expanded (Fig 4A). The LL scenario had the greatest average drop in total cropland area (-12,033 km^2^), largest increase in rangelands (+8,952 km^2^) and lowest amount of new development (+3,081 km^2^). Conversely, the HH scenario had the greatest increase in developed land (+14,720 km^2^), highest perennial cropland expansion (+6,826 km^2^), and was the only scenario to show overall expansion of total cropland area (+1,815 km^2^). Rangelands decline dramatically in all of the “high” scenarios (-6,114 < > -16,536 km^2^) and in the BAU (-4,712 km^2^), increasing only in the LL scenario (+8,952 km^2^; Fig 4B). Overall, the LL and HH scenarios represent the outer bounds of all scenarios in terms of land use extremes, with the remaining 5 scenarios falling somewhere between their range.

**Fig 5:**
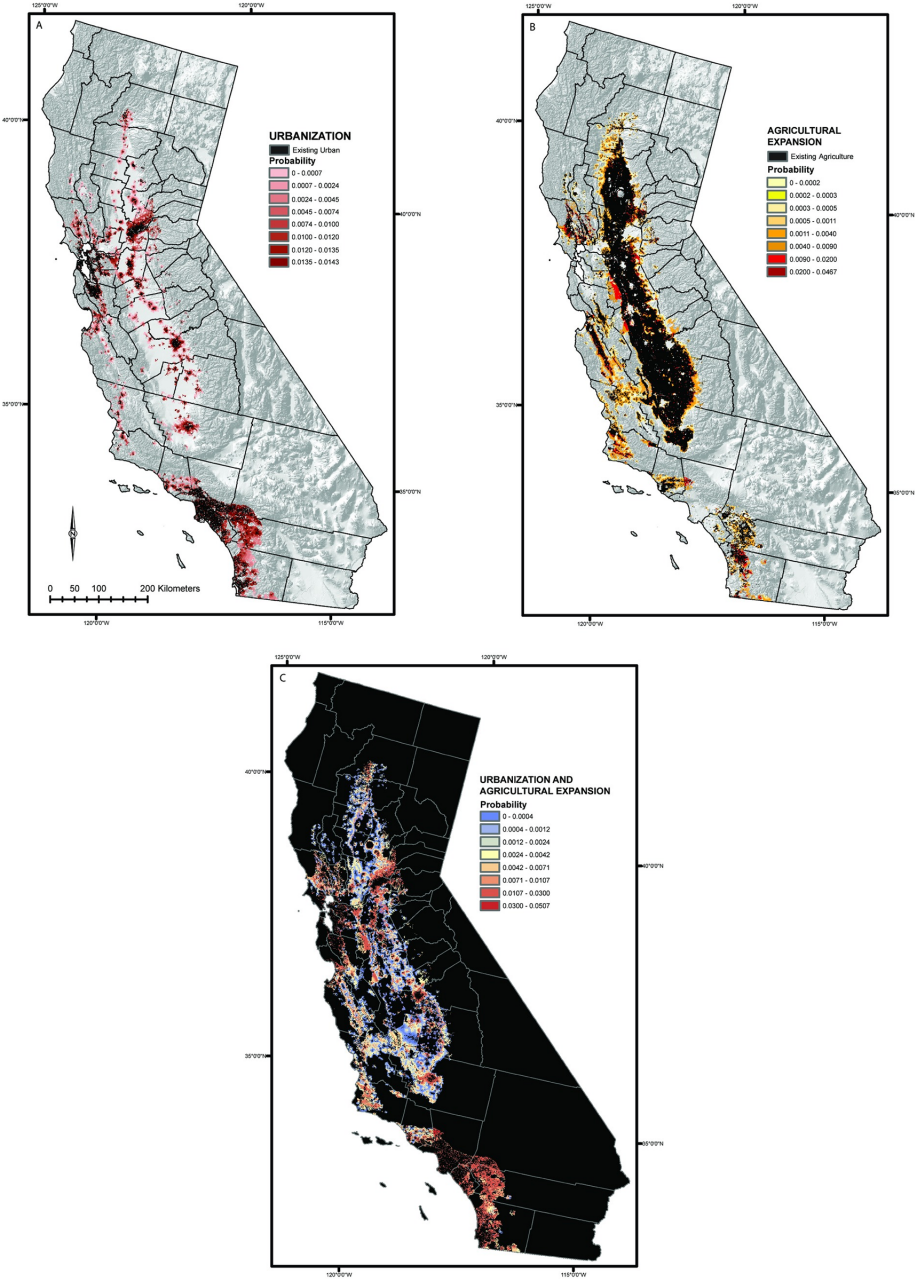
Annual transition probabilities across all seven scenarios for urbanization and agricultural expansion from 2012–2062. (A) Average annual transition probability for urbanization across all scenarios. (B) Average annual transition probability for agricultural expansion across all scenarios. (C) Cumulative average annual transition probability for conversion to anthropogenic land uses (urbanization and agricultural expansion). Shaded relief map from the U.S. Geological Survey’s National Atlas of the United States [64].

Combining the spatial LULC change results across all seven scenarios provides a clear visualization of the most probable future change areas. The model produced average annual transition probability maps over the 70 year (1992–2062) simulations for each scenario. The annual transition probability maps from all seven scenarios were then averaged for development (Fig 5A) and agricultural expansion (Fig 5B) to highlight areas most likely to undergo these transitions over the simulation period and across scenarios. Most new development was projected around existing developed areas near Sacramento and in southern California, while agricultural expansion was most likely near existing agricultural land. Fig 5C is the summed cumulative average transition probability of both urbanization and agricultural expansion highlighted areas likely to undergo some form of land use intensification over the model period.

### State-wide water use

**Fig 6:**
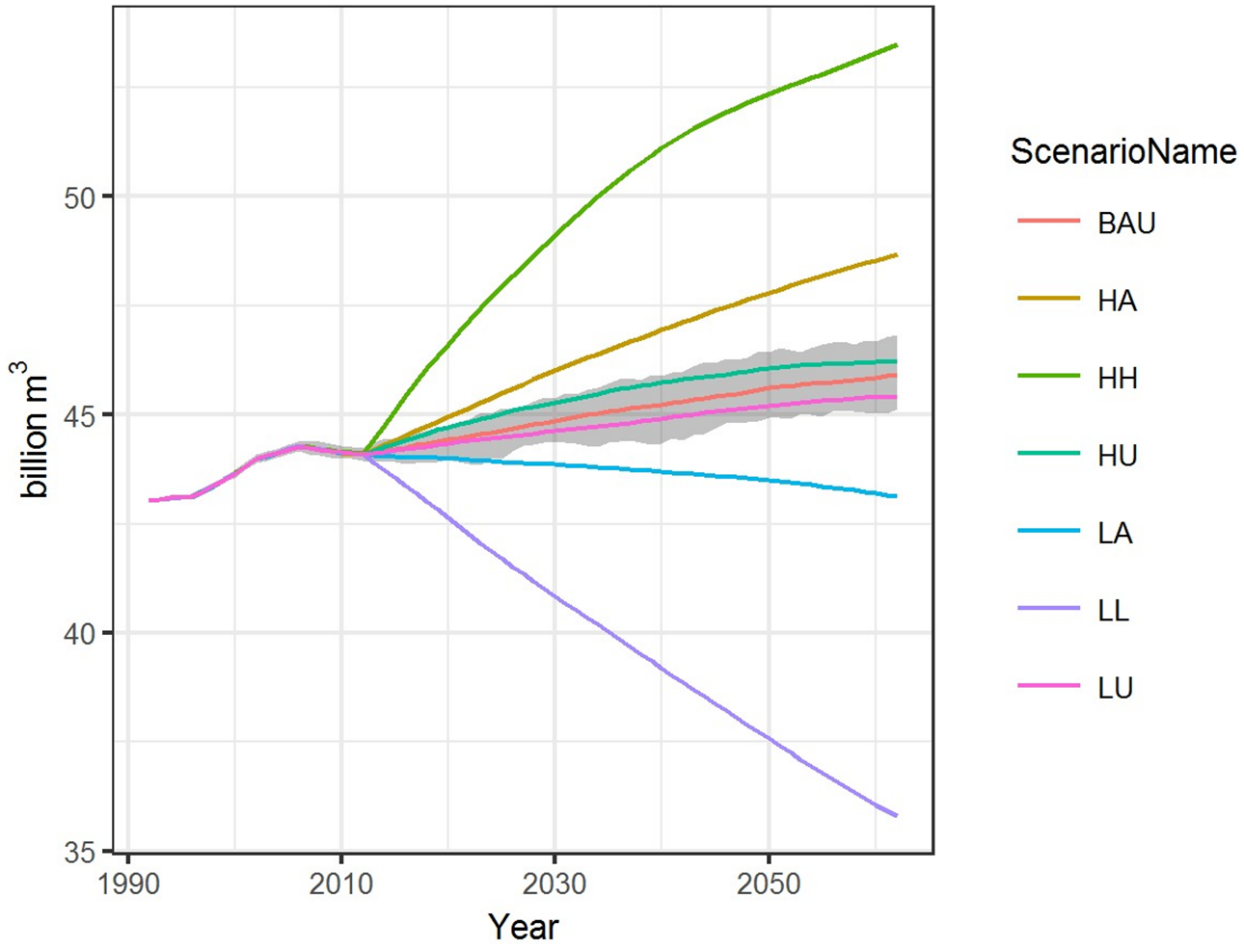
Average net change in water use demand across scenarios from 1992–2062 across scenarios. Net water use demand values expressed in billions of cubic meters (10^9^ m^3^) across seven future land use and land cover (LULC) scenarios for Mediterranean California. Shaded area shows the maximum and minimum value ranges across 40 Monte Carlo simulations for the BAU scenario only.

Water demand by 2062 was projected to increase over 2012 values in the BAU and all but the LA and the LL scenarios (Fig 6). The LU scenario showed the lowest overall increase at ~ 1.3 billion cubic meters (Bm^3^), followed by the BAU (+1.8 Bm^3^), HU (+2.1 Bm^3^), HA (+4.6 Bm^3^), and HH (+9.4 Bm^3^). Between 2012 and 2062, this equates to an average 3.0–5.0% overall increase for the LU, BAU, and HU scenarios and 10.4% (HA) and 21.2% (HH). Water demand dropped 18.8% in the LL scenario (- 8.3 Bm^3^) and a modest 2.2% in the LA scenario (-0.9 Bm^3^).

**Fig 7:**
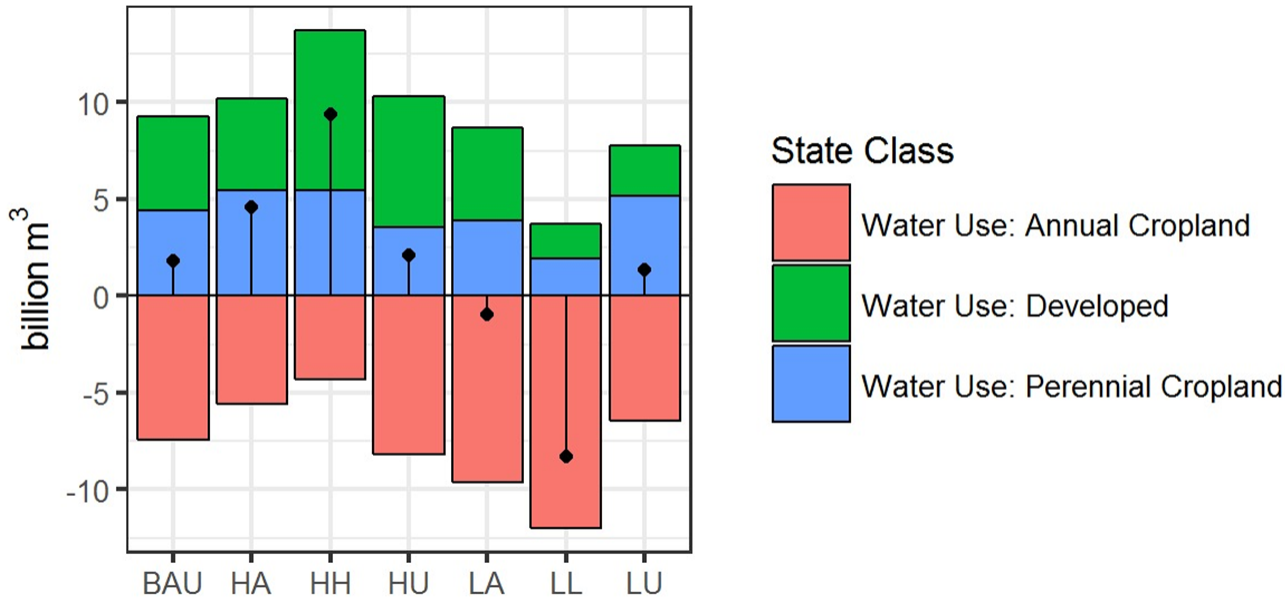
Average net change in water demand (2012–2062) for each scenario by land use type. Values for annual cropland (red) perennial cropland (blue) and developed (green) with black line indicating average net change summarized for all land use types across 40 Monte Carlo simulations.

Water demand change by land use type (Fig 7) shows water use for annual cropland declining across all scenarios and increasing for both perennial cropland and development. Across scenarios, annual cropland was projected to use between -4.3 and 12.0 Bm^3^ less water by 2062, while perennial cropland increased between 1.9 and 5.5 Bm^3^ and developed water demand increased between 1.7 and 7.9 Bm^3^. Overall, annual cropland water demand decreased 17.8% in the HH scenario and from ~ 40 to 50% in the LA and LL scenarios, respectively. Perennial cropland water demand increased from 32.8% in the LA scenario to 43.0% in the LU, and approximately 45% in both the HA and HH scenarios. In the LL and LA scenarios, water demand for perennial cropland was projected to surpass annual cropland water use by 2062 by 1.6 Bm^3^ and 1.2 Bm^3^ respectively. Developed water use increased across all scenarios, more than doubling in the HH scenario (+8.2 Bm^3^) and increasing over 85.5% (+6.7 Bm^3^) in the HU scenario. Cropland total use by 2062 ranged from 26.1 to 37.3 Bm^3^ while developed land uses account for between 9.6 and 16.1 Bm^3^ total water demand.

### County-level and regional water use

**Fig 8:**
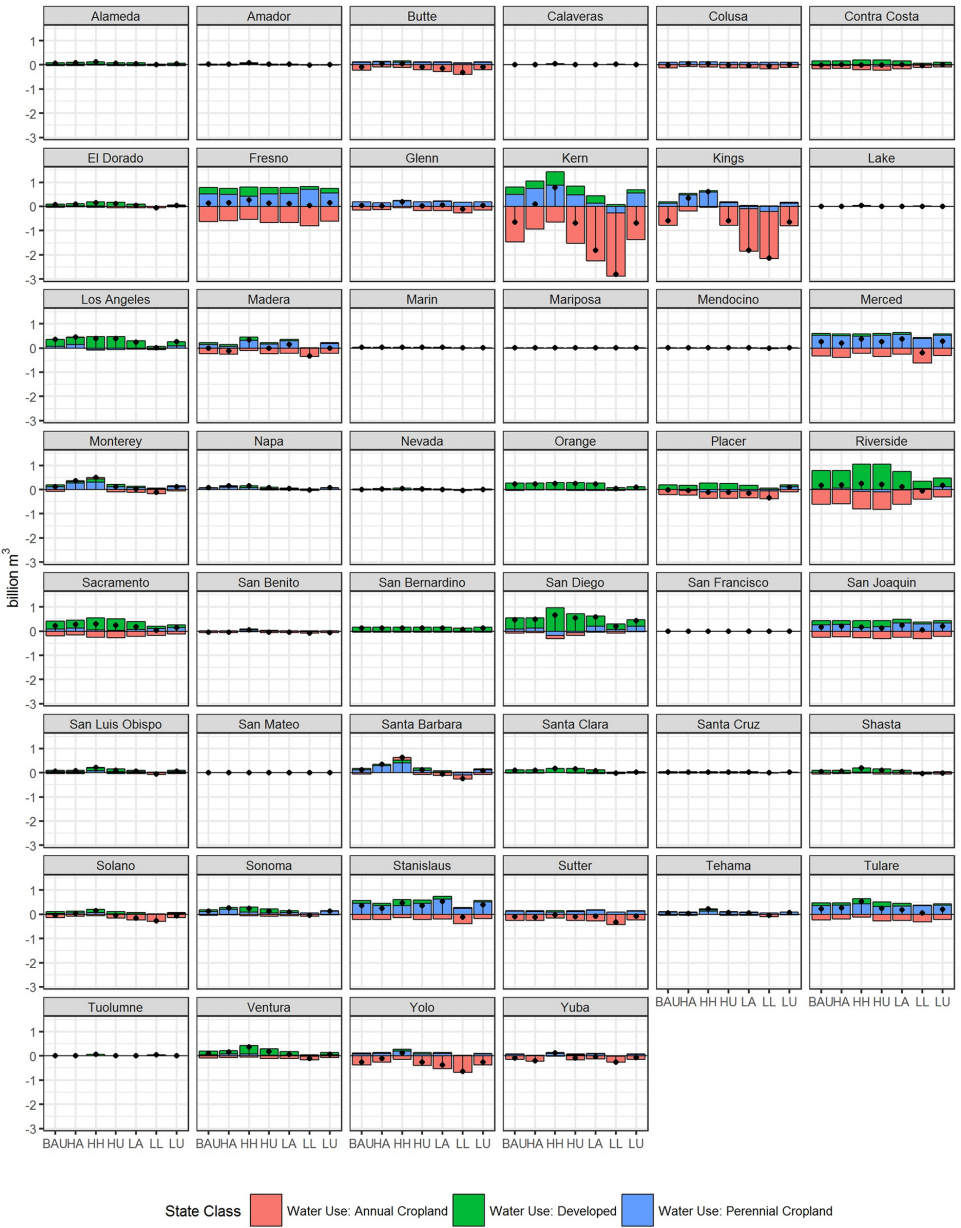
Projected net change in water demand by county and scenario. Net change in water use demand (1992–2062) in billions of cubic meters (Bm^3^) for each county for annual cropland (red), perennial cropland (blue), and developed (green) land uses. The black dot indicates average net change value.

Overall projected water demand in many counties increased across all scenarios by 2062, with the greatest increases in Los Angeles, Sacramento, and San Diego Counties. Counties in southern California showed the largest increased demand for developed water use (i.e. Los Angeles, Orange, Riverside, San Bernardino, and San Diego Counties) along with Sacramento County, with modest increases in the San Francisco Bay Area (i.e. Alameda, Contra Costa, and Santa Clara, and Ventura Counties) (Fig 8). In all cases, annual cropland water demand declined, with the greatest declines occurring in Kern, Fresno, Kings, and Riverside Counties. Expansion of perennial cropland led to increased water use in Fresno, Kern, Merced, Stanislaus, and Tulare Counties. Shifting cropland demands drive overall change in water demand with the greatest declines in Kern and King Counties. Tulare, Merced, San Joaquin, Stanislaus, and Fresno Counties each show increased water demand driving primarily by perennial cropland expansion.

**Fig 9:**
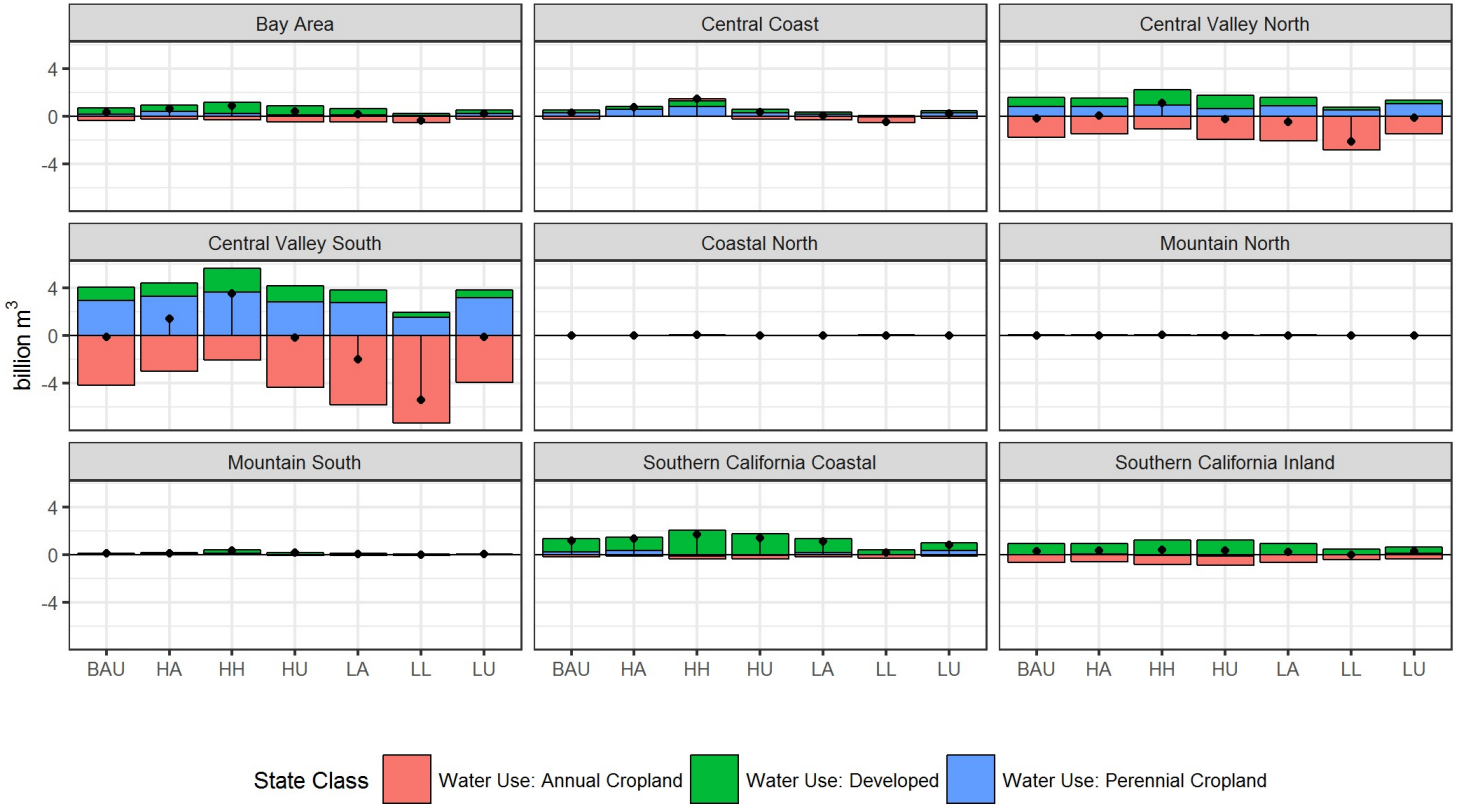
Projected net change in water demand by geographic region. Net change in water use from 1992–2062 for each land use category by geographic region (colored bars) and total (black points) under each scenario.

At the regional level, counties in the Central Valley South showed the greatest variability to future water demand and also have lower resilience to drought and decreased supply (Fig 9). The region is characterized by low annual runoff coupled, high rainfall variability, and non-local water deliveries [79], as well as high rates of fallowing in dry years [15]. Perennial cropland expansion drives increased water demand here, coupled with smaller increases in water demand for development and significant declines in annual cropland. A similar, yet smaller magnitude pattern emerged for the Central Valley North region. The Coastal North, Mountain North, and Mountain South regions show limited change across scenarios, as land use demand in these areas is comparatively low. The Bay Area and Southern California Coastal region show increased demand for expanding development.

## Discussion

This research demonstrates a methodology for creating land use scenarios based on historical land change and water use information and varying assumptions of future land use. Land use scenarios are powerful tools for analyzing potential future landscapes and resource use outcomes and visualizing the consequences of future potential policy/land use decisions. While these scenarios are in no way predictions nor should they be considered absolute outcomes, they do represent a wide range of plausible future outcomes in terms of land-use related water demand, based on historical trends. The seven scenarios also enable examination of how alternative future land use intensities and potential land use policies might influencer future water demand.

The findings presented here reveal several key issues for California land and resource managers. First and foremost, future agricultural land use will continue to be the single largest driver of water demand, continuing its present trend as California’s dominant consumer. Despite all scenarios demonstrating some degree of annual cropland decline, continued expansion of perennial cropland and development makes up of for these losses. It is unclear how new regulations or future policy may alter this outcome, yet passage of the 2014 Sustainable Groundwater Management Act may further test the agriculture’s resiliency to drought, as the regulation will place limits on groundwater over-drafting, a common occurrence during dry years. Expansion of developed lands does lead to overall changes in the proportion of agricultural water demand, dropping from ~82% of all demand in 2012 to between 68–77% by 2062 across scenarios. Only in the low agriculture (LA) and the lowest of the low (LL) scenario did future water demand decline over time. In all but the lowest of the low (LL) scenario, rangelands (i.e. grasslands and shrublands) see consistent losses, dominated primarily by new developed land uses and to a lesser extent by agricultural expansion. This has important implications for future groundwater recharge, changes in surface drainage, carbon sequestration, ecosystem connectivity, habitat, grazing, open space and recreation [51].

Our new business-as-usual (BAU) average water demand change findings of 1.814 Bm^3^ compare favorably with the 1.817 Bm^3^ results previously published [56]. This demonstrates the reproducibility of model results and helps further validate model performance. The previous BAU scenario used transition targets randomly sampled from the historical distribution outside of the model for each year and Monte Carlo, while the new BAU scenario randomly sampled from the historical distribution iteratively and directly within the LUCAS model framework—a new and improved model function. The low agriculture scenario (LA) scenario samples from years where agricultural contraction exceeded agricultural expansion and overlaps an extended, near state-wide drought period (2007–2010) and thus represents a multi-decadal drought scenario. Both the HH and LL scenarios represent extremes in terms of land use outcomes. Given the spatial and temporal dis-aggregation of these two scenarios (i.e. sampling from different years for low and high values for each county) the relative land change drivers which might lead to the HH and LL outcomes cannot be determined as they are independent in both space and time. They do, however, effectively create upper and lower bounds of all possible futures based on county level historical extremes.

Several improvements can be made in future work modeling water use demand. An improved representation of cropland types, such as vineyards and orchards, would better represent regional variability in water use. These crops have different water use requirements and irrigation strategies. Such refinement would be an improvement on the spatially weighted average water use approach used in the current analysis. Our work also assumes the trend in high value cash crops will continue indefinitely. Making predictions about changes in diet, market factors, and social preferences were beyond the scope of the current work. The scenarios presented here also do not account for future changes in water availability and individual water use, improved efficiencies, or changes in urban density over time. Incorporating population data and per capita water use would greatly improve water demand estimates for municipal developed land uses.

We did not incorporate future climate in our scenarios which would provide a more informed look at land use options and changes in available water supply. Future climate will likely be an important driver of individual land use decisions, especially in the farming sector, as water allocations often drive cropland choice. Our low agriculture scenario does follow trends seen in historical years where drought necessitated extensive fallowing of otherwise productive land and agricultural contraction, mirroring a prolonged drought future. Farmers often turn to groundwater in times of drought as well. Nearly 9% of all watersheds in the drought year 2013 were water stressed, mostly driven by agricultural demand except for in Southern California where development-related water use dominated demand [80]. New management of previously unmonitored groundwater use may trigger use restrictions during drought which could impact cropland choice, water pricing, and cropland expansion rates. Analysis of future climate driven changes in surface water shows the entire state of California having a 5–25% increase in climate-induced water stress (i.e. where demand outpaces natural supply) by mid-century over 1900–1970 levels [80]. Persistent drought may also impede hydroelectric power generation in years with low reservoir storage as thermoelectric power generation drops as much as 16% in summer due to water shortages [81].

Long-term water management planning should take into consideration highly variable annual rainfall, drought periodicity, and future projected climate change when factoring water availability and deliveries across multiple years. A single wet winter should not become the basis for relaxed conservation, given the recent extreme drought and long-term drought projections this century [12]. In fact, as Governor Brown’s Executive Order B-37-16 makes clear, water conservation should be a way of life in California [82]. Any expanded need for water will also bring with it increased energy use for storage, transport, and delivery. The water sector in California currently uses nearly 20% of all the electricity in the state and 30% of the natural gas [83]. Therefore, future water-related energy consumption and energy related water consumption (i.e. water-energy nexus) should also be a cause for concern. The majority of the rainfall in California is received and stored in the north and transported southward to users. Southern California also depends on water delivered from the Colorado River which may be further depleted in coming decades as rising municipal demand outpaces average supply [84]. Given most of our modeled increases in water demand occurred in the southern part of the state, any increased water demand will increase greenhouse gas emissions from power usage, a negative feedback for climate change.

Water use in California will inevitably rise in coming decades. Given the historic variability in annual precipitation, future projected climate warming, population growth in the state and globally, and increased demand for agricultural products, any additional demand for water will likely stress existing supplies. With the 7^th^ largest economy in the world, global shifts in demand for food, fiber, and energy will continue to place pressure on California’s landscape. Nearly every new resource demand relies, to some extent, on water availability, from hydroelectric power generation and irrigation to industrial and municipal consumption. The recent almond boom, for example, resulted in an estimated 27% increase in irrigation demand between 2007 and 2014 alone [85]. Our research demonstrates that under current water use efficiencies and rates of anthropogenic land use, water demand in California will likely continue to increase. California’s resiliency to increased water demand during water limited periods will likely depend on the type and intensity of agriculture, per capita and industrial consumption, water use efficiency and conservation, as well as improved management of all water resources.
